# Draft genome sequence of a prodigiosin-hyperproducing *Serratia marcescens* strain isolated from Cairo, Egypt

**DOI:** 10.1093/g3journal/jkab284

**Published:** 2021-08-06

**Authors:** Nora M Elkenawy, Noha H Youssef, Ramy K Aziz, Magdy A Amin, Aymen S Yassin

**Affiliations:** 1 Drug Radiation Research Department, National Center for Radiation Research &Technology, 11787 Cairo, Egypt; 2 Department of Microbiology and Molecular Genetics, Oklahoma State University, Stillwater, OK 74078-5061, USA; 3 Department of Microbiology and Immunology, Faculty of Pharmacy, Cairo University, 11562 Cairo, Egypt; 4 The Center for Genome and Microbiome Research, Cairo University, 11562 Cairo, Egypt; 5 Microbiology and Immunology Research Program, Children’s Cancer Hospital Egypt 57357, 11617 Cairo, Egypt

**Keywords:** *Serratia marcescens*, prodigiosin, bioinformatics, genome annotation, genome assembly

## Abstract

*Serratia marcescens* is a Gram-negative bacterium with both environmental and host-associated strains. Pigmentation is reportedly inversely correlated with infection frequency, and prodigiosin is one of *Serratia* pigments that has medical and industrial applications. Here, we report the draft genome sequence of prodigiosin-hyperproducing *Serratia marcescens* strain N2, isolated from Cairo, Egypt. The sequence is assembled into 142 contigs, with a combined size of 5,570,793 bp. The assembled genome carries typical *S. marcescens* genes, with potential prodigiosin-biosynthesizing genes detected.

## Introduction

*Serratia marcescens* is a Gram-negative rod-shaped bacterium, belonging to family *Enterobacteriaceae*. It has been isolated from various environmental and nosocomial sources. In the last decades, *S. marcescens* has been recognized as a significant opportunistic human pathogen, as it was found responsible for a variety of symptoms in hospitalized patients, including septicemia, meningitis, and infections of the urinary tract (Abreo and Altier 2019). Although some *S. marcescens* strains are associated with hospital infections, pigmented *S. marcescens* strains were shown to cause infections in a much lower frequency than nonpigmented strains. This observation implies that the infection risk is minimal during the mass production of pigment (Roy *et al.* 2014). 

Prodigiosin is a red pigment produced as a secondary metabolite by *S. marcescens*, characterized by a distinctive tripyrrole structure, responsible for its reported multiple pharmacological effects as anti-cancer, anti-microbial, anti-oxidant, and immunosuppressant, as well as its unique application as a natural dye for olefins and textiles (Lin *et al.* 2019). Factors such as temperature, pH, dissolved oxygen levels, light and medium composition influence the production of prodigiosin (Aruldass *et al.* 2014). Here, we report the genome sequencing of *S. marcescens* strain N2, which was isolated from a local hospital in Cairo, Egypt. This strain is capable of producing 870 unit/cell of prodigiosin after 6 days of incubation (Elkenawy *et al.* 2017).

## Materials and methods

### DNA extraction

Genomic DNA was extracted by the PrepMan^®^ ultra sample preparation reagent (Applied Biosystems, USA). Phylogenetic identity was confirmed by 16S rRNA gene sequencing, performed by the MicroSeq^®^ 500 identification protocol (Applied Biosystems, USA), as previously described (Fontana *et al.* 2005).

### Sequencing

The genome of *S. marcescens* strain N2 was sequenced on an Illumina MiSeq platform at Novogene (Beijing, China), following the standard Illumina protocols. A 300 × 2 paired-end chemistry was used, with an average library insert size of 700 bp. The Nextera XT DNA library prep kit (Illumina, San Diego, CA, USA) was used for the preparation of sequencing libraries from extracted DNA, as per the manufacturer’s instructions.

### Preprocessing and assembly

Reads were quality-filtered by Trimmomatic, version 0.36 (Bolger *et al.* 2014), with adaptor clipping option, a sliding window size of 4 and a sliding window minimum quality of 15. High-quality reads were assembled by three different tools: (1) IDBA assembler (v1.1.3) with minimum contig length of 2000 bp (Peng *et al.* 2012); (2) SPAdes assembler (v3.12.0), which is part of the Pathosystems Resource Integration Center (PATRIC) services (Antonopoulos *et al.* 2019; Davis *et al.* 2020), with minimum contig length of 500 bp; and (3) Unicycler assembler (v0.4.8), within PATRIC services as well, with minimum contig length of 500 bp (Table 1).

### Genome annotation

The assembled genome was annotated by PATRIC (version 3.6.9) annotation service (Antonopoulos *et al.* 2019; Davis *et al.* 2020), which uses the RASTtk algorithm (Aziz *et al.* 2008; Brettin *et al.* 2015).

## Results and discussion

The sequencing yielded 13.3 Gb of paired-end reads, with an average read length of 150 bp.

All three genome assemblers produced high-quality contigs, ranging in number from 142 to 716, and an N50 from 41,216 to 238,944 (Table 1). Based on the comparison between the three assemblers, Unicycler assembly was further considered for downstream annotation and analysis steps. The 142 contigs had a combined size 5,570,793 bp with an *N*_50_ of 238,944 bp, an *L*_50_ of 8, and a G + C content of 59.05%.

**Table 1:** The reeads assembly using three different tools

	IDBA	SPAdes	Unicycler
Total *N* contigs*a*	271*a*	716	142
*N* contigs >1,000	271	195	112
*N* contigs >10,000	127	55	44
Largest contig	201,798	405,373	532,301
*N*_50_	41,216	171,039	238,944
GC%	56.26	58.32	59.05
Total length	5,360,385	5,796,868	5,570,793

a IDBA used a threshold of 2000 bp. as a minimum for assembled contigs to be included, while SPAdes and Unicycler used a threshold of 500 bp. This made the total number of contigs equal to the number of contigs >1,000 in IDBA assembly.

Genome annotation resulted in 5615 coding sequences (CDS), 86 tRNAs and 6 rRNA-encoding genes. The CDS included 1200 hypothetical proteins and 4415 proteins with functional assignments, of which 2064 proteins were assigned EC number, 1068 were assigned gene ontology (GO) terms, and 1471 proteins were assigned to known pathways. PATRIC assigned the proteins to 5024 genus-specific families (PLfams) and 5170 cross-genus families (PGfams). The assembled genome was also analyzed by NCBI prokaryotic genome annotation pipeline service (Tatusova *et al.* 2016), which is usually more conservative in gene calling and functional assignments. NCBI analysis predicted 5393 CDS, a number that is closest to the PATRIC genus-specific families. BLAST identified proteins that form clusters for prodigiosin production, including PigA, PigB, PigC, PigD, PigE, PigF, PigH, and PigI (Williamson *et al.* 2006). Similarly, transcriptional regulators for pigment production were also identified, *e.g.*, PigS (Positive Transcription regulator), PigT, HexS (Direct downregulator), CRP (Indirect downregulator), PigP (Transcription activator), PigX (Transcription repressor), and Rap (Activation regulator of antibiotic and pigment) (Gristwood *et al.* 2011).

Finally, the use of PATRIC similar genome finder (Antonopoulos *et al.* 2019), which uses the Mash/MinHash algorithm (Ondov *et al.* 2016) predicted the closest genome neighbor to strain N2 to be *S.* *marcescens* strain ATCC 274 (NCBI accession number: AP021873, RefSeq ID: NZ_AP021873). Further analyses of the genome sequence, coupled with high-throughput techniques such as random transposon mutagenesis (*in vitro*) and genome-wide metabolic reconstruction (*in silico*), will enable the identification of genes putatively involved in prodigiosin promotion and provide more insight into the biosynthesis and regulation of structurally diverse secondary metabolites.

## Data availability

Raw reads are available under SRA accession SRX10696011. The whole-genome shotgun project has been deposited at DDBJ/ENA/GenBank under Bioproject ID PRJNA525074, Biosample ID SAMN11041520, and WGS accession SPSG00000000. The version described in this paper is version SPSG02000000. The annotated genome the PATRIC database is deposited under genome number 615.1488.
